# Supplementary material for: FUS-dependent loading of SUV39H1 to OCT4 pseudogene-lncRNA programs a silencing complex with OCT4 promoter specificity
Source: Commun Biol. 2020 Oct 30;3:632. doi: 10.1038/s42003-020-01355-9 (PMC7603346; doi:10.1038/s42003-020-01355-9)
Supplement: Supplementary file 1 — Supplementary Information [file 42003_2020_1355_MOESM1_ESM.pdf]

Supplementary information file

FUS-dependent loading of SUV39H1 to *OCT4* pseudogene-lncRNA programs a silencing complex with *OCT4* promoter specificity

Michele Scarola[1,3], Elisa Comisso[1,3], Massimo Rosso[1, 2],

Giannino Del Sal[1, 2], Claudio Schneider[1, 3], Stefan Schoeftner[1, 2,*] and Roberta Benetti[1, 3,*]

1. Laboratorio Nazionale - Consorzio Interuniversitario per le Biotecnologie, Laboratorio Nazionale (LNCIB), Padriciano 99, 34149 Trieste, Italy.
2. Dipartimento di Science della Vita, Università degli Studi di Trieste, Via E. Weiss 2, 34127 Trieste, Italy
3. Dipartimento di Area Medica (DAME), Università degli Studi di Udine, p.le Kolbe 1, 33100, Udine, Italy

[*] Corresponding authors: e-mail: sschoeftner@units.it; e-mail: roberta.benetti@uniud.it

a

OVCAR-3 cells	Molecules / µg RNA	Absolute fold (*DAXX* set as 1)
hOCT4P3 lncRNA	78600	1 : 129,77
OCT4 mRNA	67200	1 : 151,78

b

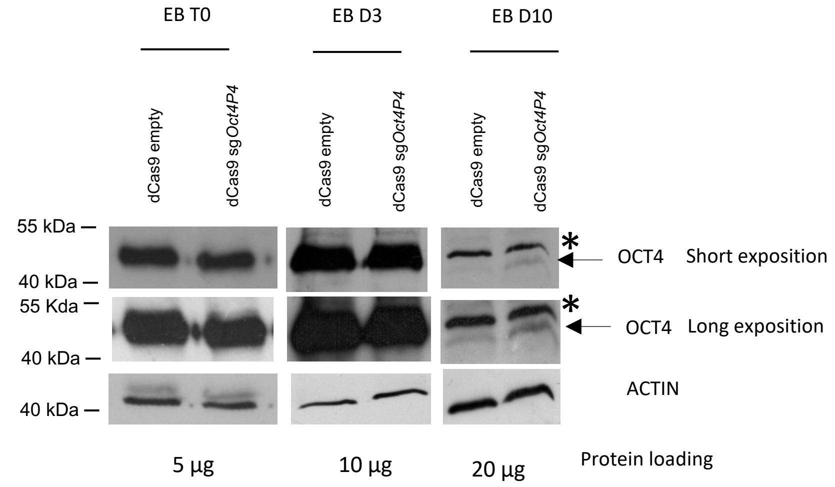

c

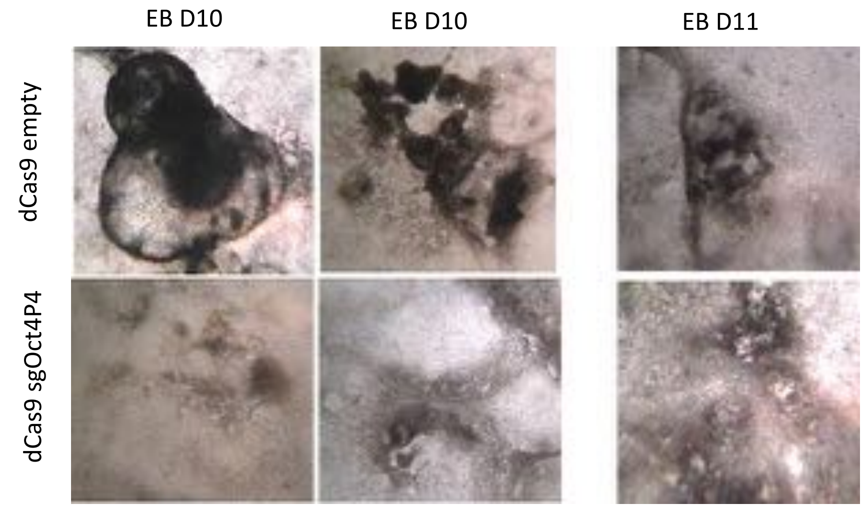

(a) Quantitative assessment of *hOCT4P3* and *OCT4* transcripts in experimental OVCAR-3 cells as determined by quantitative RT-PCR (see supplementary material and methods). Number of *hOCT4P3* and *OCT4* transcripts in 1µg of total RNA are indicated; proportion of *hOCT4P3* and *OCT4* transcript levels to the reference transcript *DAXX* are shown. **(b)** Western blotting of undifferentiated mESCs (EB T0) and embryoid body cultures after 3 (EB D3) or 10 (EB D10) days of *in vitro* differentiation. Used antibodies are indicated. **(c)** Embryoid body structures of control (dCas9 empty) and dCAS9 sgOct4P4 ES cells after 10 or 11 days of differentiation.

Supplementary Figure 1

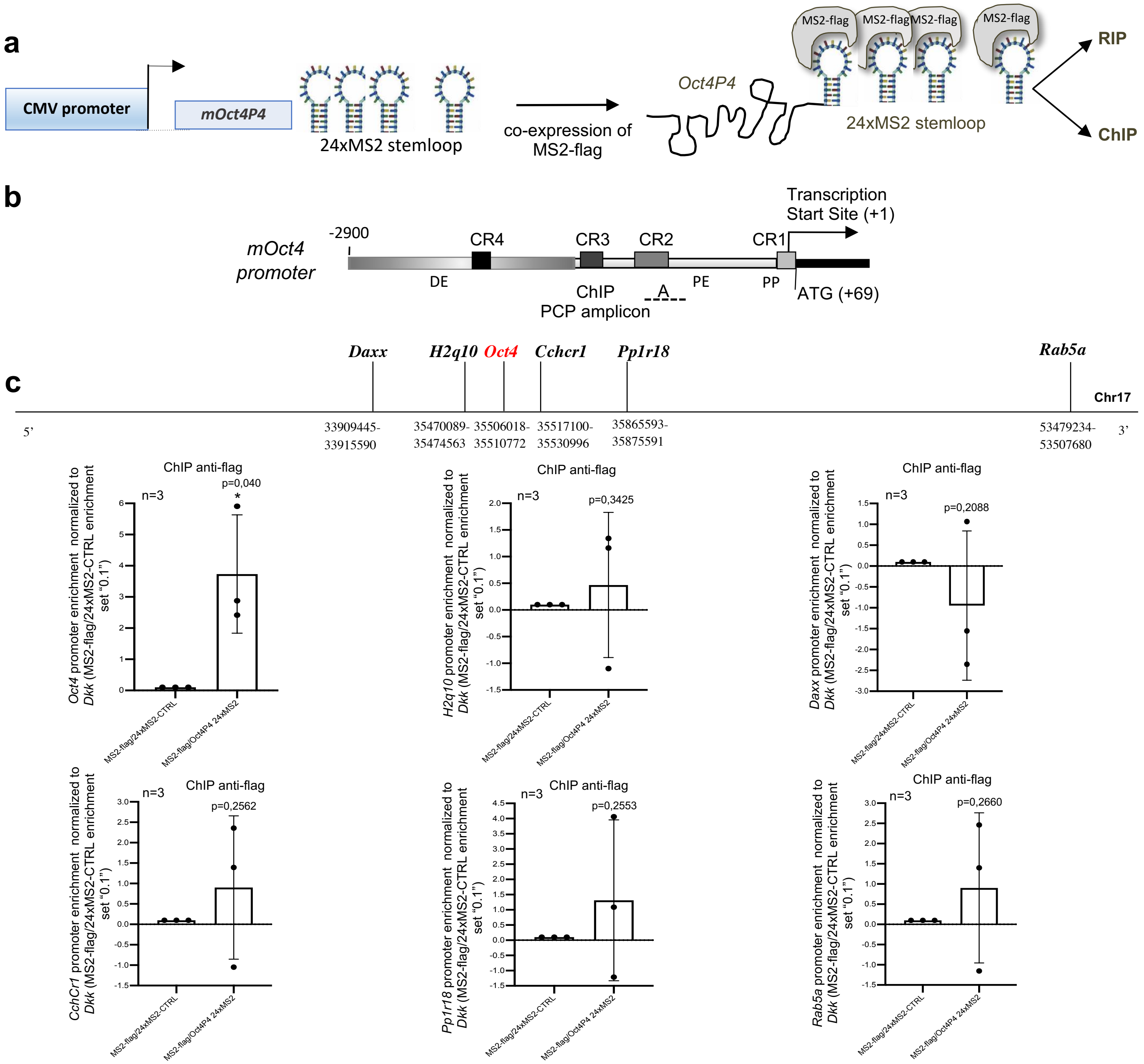

d. **200bp-*mOct4P4* (343 bp)**

GTCCCTAGGTGACCAACTCCTCCTTCTGTCAGGCTCCCAACTTGGGGTGCCCAACTTCCCCGCGGCCCCTGCTGGAGAAG
TGGGTGGAGGAAGCGGACAACAATGAGGATGTTCAAGGATGTATTGCAAACCCTGGTGTAGGCCTGCAAGAGAAAGCAGA
TGAGCATTGAGAACCCTGAGAAGGGGAACCTGAAAAACACATTTCTGCAGTGGCGGAAGCCTACCCTGCAGCAGGTCAGC
AGCATCATCAAACAGCTTGGGTAGAGAGGAAGATGAAGTTATGCTGTTGAGTGTGAGCTGTCACTCACCATTTTGTTCTTGA
ATTTAAAAAGCCTGGGACACA

(a) Schematic representation of the *mOct4P4-24xMS2* construct used to generate mESCs stably expressing MS2-flag and *mOct4P4-24xMS2* lncRNA. **(b)** Schematic representation of the mouse *Oct4* promoter region. The location of the specific distal enhancer (DE), proximal enhancer (PE), proximal promoter (PP) sites and the conserved promoter/enhancer regions (CR1, CR2, CR3, CR4) are indicated. The location of the PCR amplicon (A) used in ChIP experiments is shown. **(c)** Chromatin immunoprecipitation (ChIP) analysis on promoters of genes flanking the *OCT4* gene on mouse chromosome 17. Top panel, localization of the *Daxx, H2q10*, *Cchcr1*, *Pp1r18*, *Oct4* and *Rab5a* genes; nucleotide positions are indicated. Bottom panels. Anti-flag chromatin immunoprecipitation (ChIP) analysis on indicated gene promoters in control MS2-flag and MS2-flag *mOct4P4*-24xMS2 mESCs. The unrelated *Dkk* promoter was used as a control. Error bars represent standard deviation. P-vales are inidcated; n, number of independent experiments carried out. **(d)** *mOct4P4* lncRNA sequences used to generate the 200bp-mOct4P4-24xMS2 vector. *mOct4P4* sequences corresponding to the *Oct4* UTRs are shown in black (5'UTR) and blue (3'UTR); sequence of the functional relevant 200 nucleotide *mOct4P4* region are shown in red.

Supplementary Figure 2

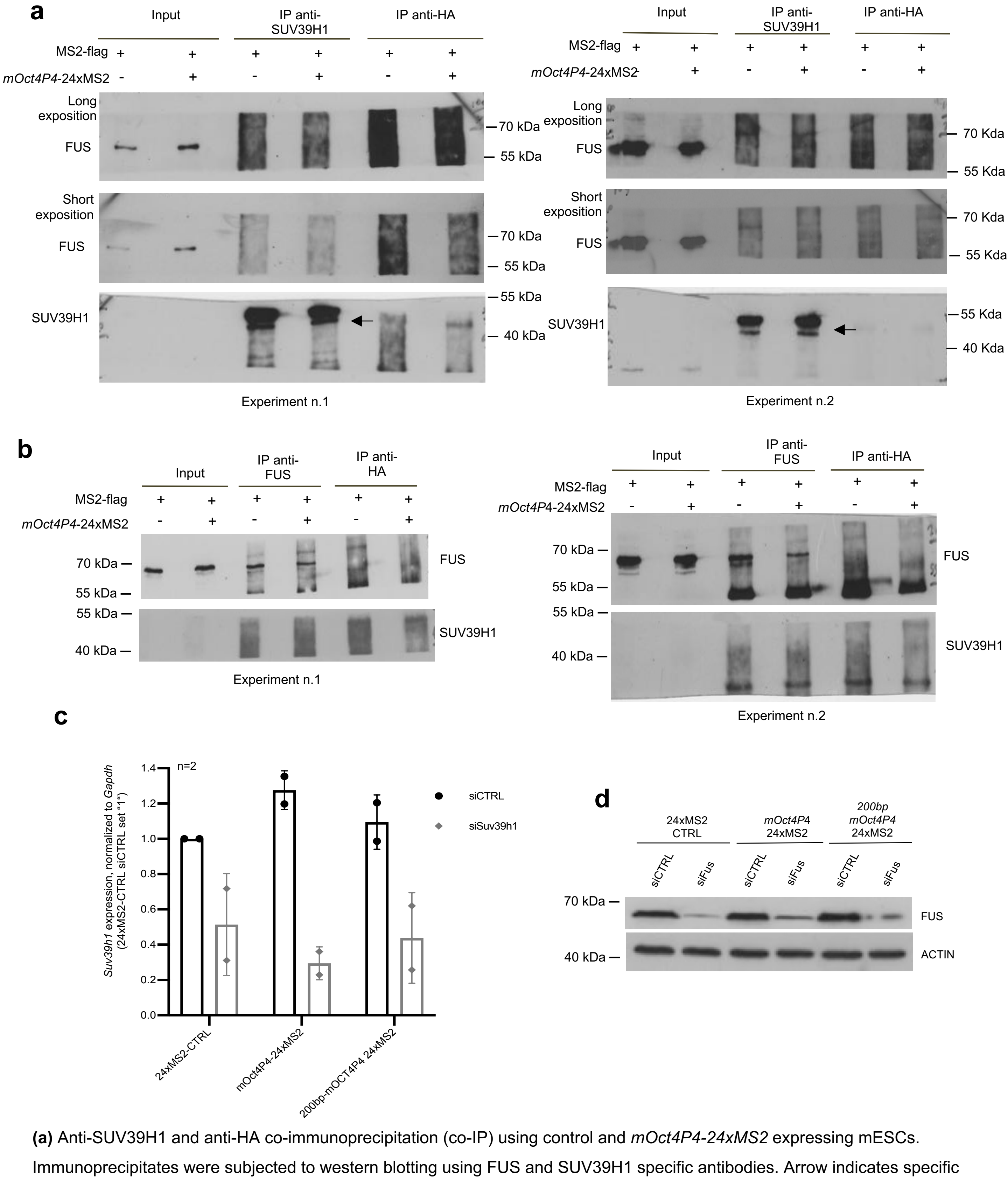

(a) Anti-SUV39H1 and anti-HA co-immunoprecipitation (co-IP) using control and *mOct4P4-24xMS2* expressing mESCs. Immunoprecipitates were subjected to western blotting using FUS and SUV39H1 specific antibodies. Arrow indicates specific SUV39H1 band. Two independent experiments are shown **(b)** Anti-FUS and anti-HA co-immunoprecipitation (co-IP) using control and *mOct4P4-24xMS2* expressing mESCs. Immunoprecipitates were subjected to western blotting using FUS and SUV39H1 specific antibodies. Two independent experiment are shown **(c)** *Suv39h1* expression in control, *mOct4P4-24xMS2* and *200bp-mOct4P4-24xMS2* mESCs transiently transfected with *Suv39h1* specific siRNA oligos, as determined by quantitative real time PCR (qRT-PCR). *Suv39h1* mRNA levels were normalized to *Gapdh*. Error bars represent standard deviation. n: number of independent experiments carried out. **(d)** Western blotting of FUS expression levels in control, *mOct4P4-24xMS2* and *200bp-mOct4P4-24xMS2* mESCs, as determined by western blotting. ACTIN was used as loading control.

Supplementary Figure 3

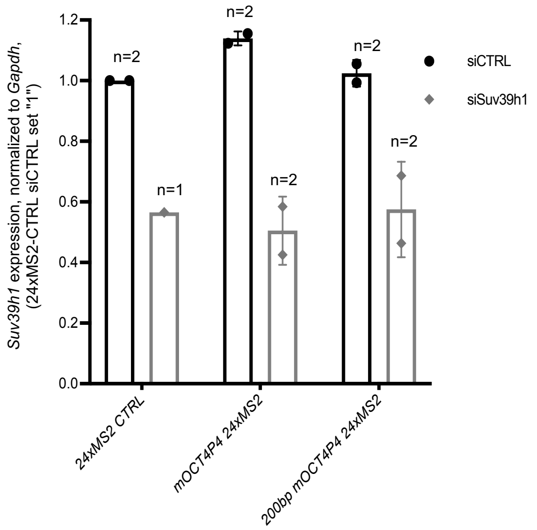

Suv39h1 expression in *mOct4P4-24xMS2* and *200bp-mOct4P4-24xMS2* mESCs under *Suv39h1* knockdown condition, as determined by quantitative real-time PCR. *Suv39h1* mRNA levels were normalized to *Gapdh*. Error bars represent standard deviation. n: number of independent experiments carried out.

a **200bp-*mOCT4P3* (530 bp)**

CAGCAAAGAACTAGAAGATATAAAGAAAAACCAAGATTTCACCATGCTTGGGGCGCCTTCCTTCCCCGCGGCCCTTG
CTGCAGAAGTGGGTGGAGGAAGCTGACAACAATGAAAATCTTCAGGAGATATGCAAAGCAGAAACCCTCGTGCAGG
CCCGAAAGAGAAAGCGAACCAGTATCGAGAACCAAGTGAGGCAACCTGGAGAATTTGTTCCTGCGGTGCCCGAA
ACCCACACTGCAGCAGATCAGCCACATCGCCCAGCAGCTTGGGGGTGCCTGCCCTTCTAGGAATGGGGACAGGG
GGAGGGGAGGAGCTAGGGAAAGAGCACCTGGAGTTTGTGCCAGGGCTTTTGGGATTAAGTTCTTCATTCACTAAGG
AAGGAATTGGAACACAAAGGGTGGGGGCAGGGGAGTTTGGGGCAACTGGTTGGAGGGAAGGTGAAGTTCAATGA
TGTTCTTGATTTTAATCCCACATCATGTATCACTTTTTTCTTAAATAAAGAAGCCTCAACACAGTAGATAGACAA

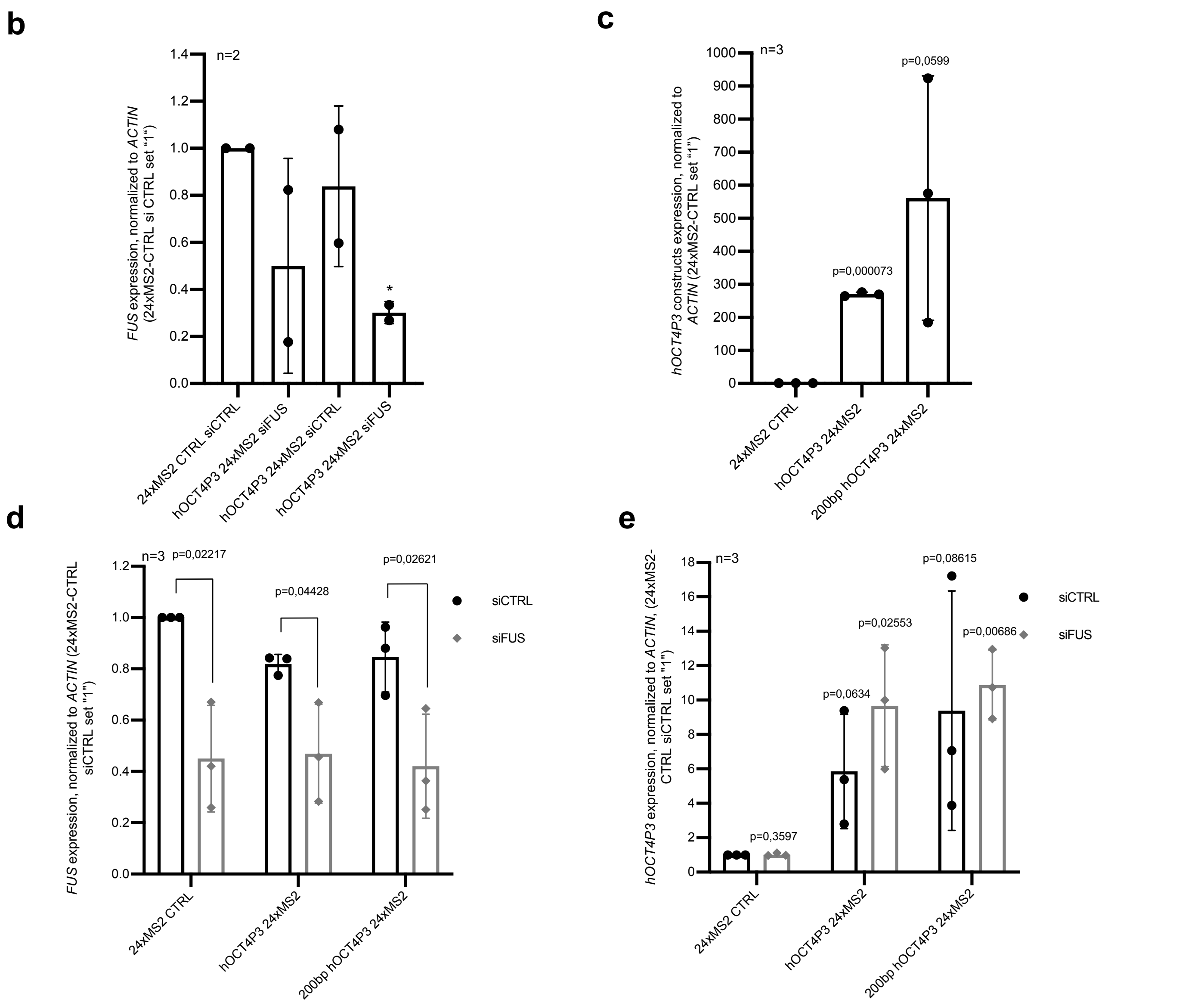

(a) *hOCT4P3* lncRNA sequences used to generate the 200bp-*hOCT4P3*-24xMS2 vector. *hOCT4P3* sequences corresponding to the *OCT4* UTRs are shown in black (5'UTR) and blue (3'UTR); sequence of the functional relevant 200 nucleotide *hOCT4P3* region are shown in red. **(b)** *FUS* expression in control and *hOCT4P3-24xMS2* expressing OVCAR-3 cells after transient *FUS* knockdown, as determined by quantitative real-time PCR. *FUS* mRNA levels were normalized to *ACTIN*. **(c)** Ectopic *hOCT4P3-24xMS2* and *200bp-mOCT4P3-24xMS2* lncRNA expression in OVCAR-3 cells stably transfected with the indicated *hOCT4P3* expression vectors. LncRNA levels were determined by qRT-PCR; *ACTIN* was used as a loading control. Expression values were normalized to *ACTIN*. Error bars represent standard deviation. **(d)** *FUS* expression in control, *hOCT4P3-24xMS2* and *200bp-hOCT4P3-24xMS2* expressing OVCAR-3 cells after transient *FUS* knockdown, as determined by qRT-PCR. *FUS* mRNA levels were normalized to *ACTIN*. **(e)** *hOCT4P3* lncRNA expression in control, *hOCT4P3-24xMS2* and *200bp-hOCT4P3-24xMS2* expressing OVCAR-3 cells after transient *FUS* knockdown, as determined by qRT-PCR. *hOCT4P3* lncRNA levels were normalized to ACTIN. Precise p-values are indicated. n: number of independent experiments carried out.

Supplementary Figure 5

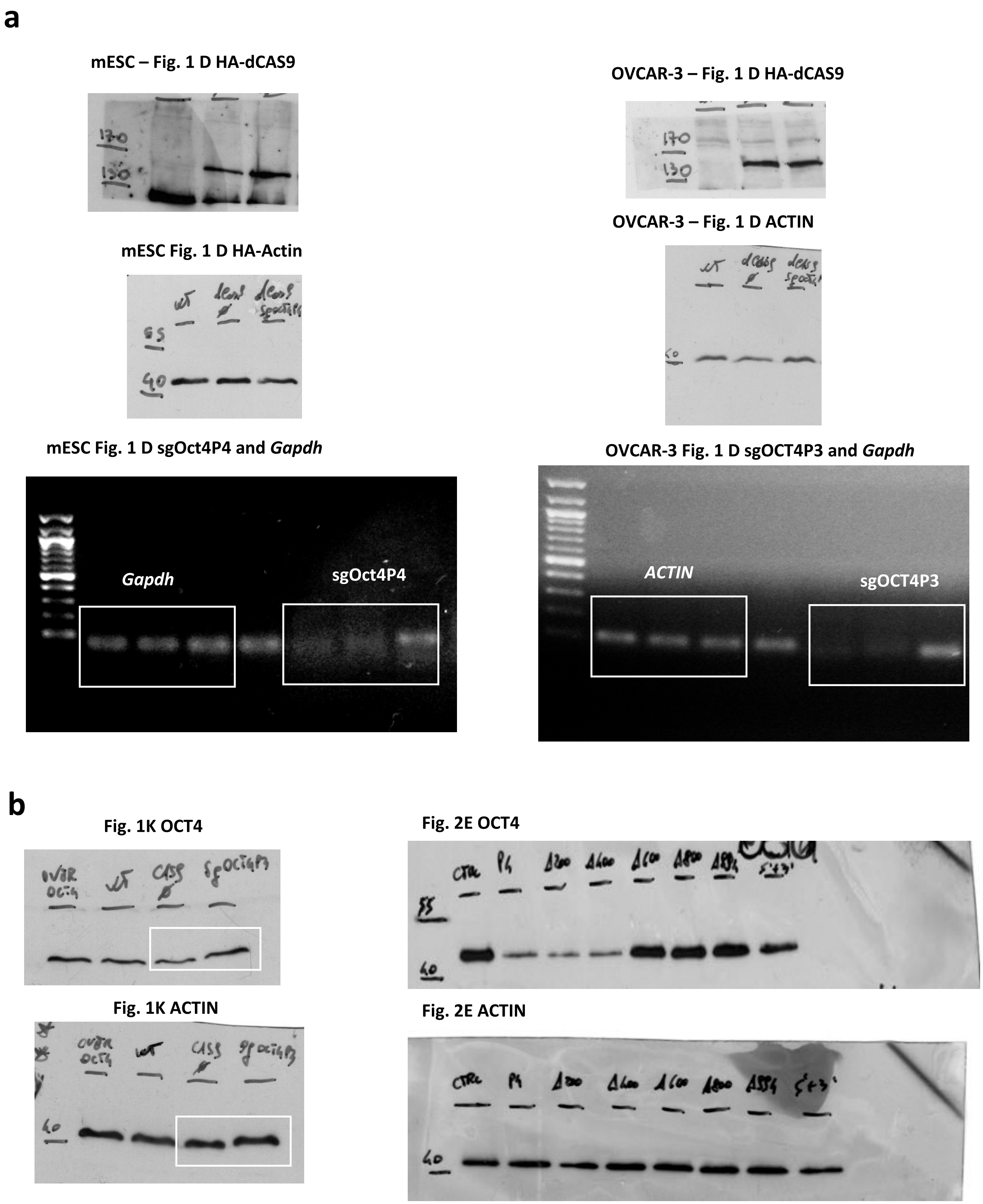

(a) Uncropped blots and gels from Figure 1. Images of the selected portions shown in Fig. 1D and Fig. 1K are indicated (b) Uncropped blots from Fig. 2.

Supplementary Fig. 6

c

Fig. 3E OCT4

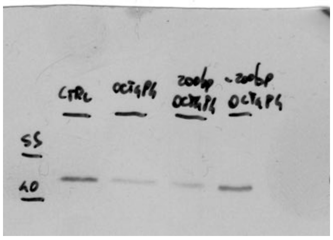

Fig. 3E ACTIN

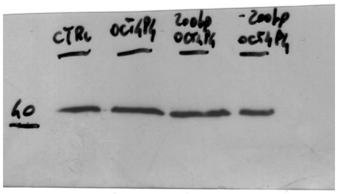

d

Fig. 4B FUS

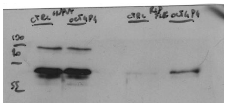

Fig. 4C FUS

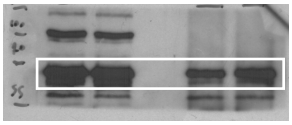

Fig. 4B MS2-flag

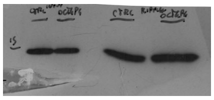

Fig. 4C MS2-flag

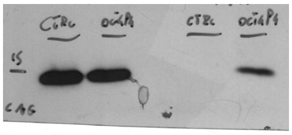

Fig. 4B *mOct4P4*

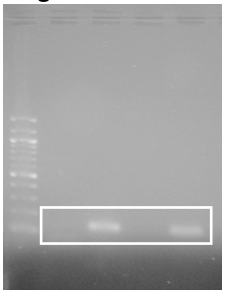

Fig. 4C *mOct4P4*

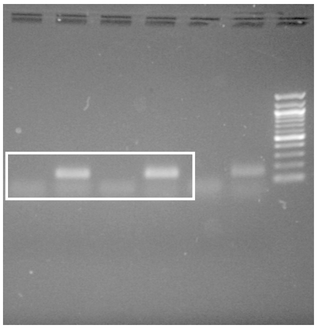

Fig. 4D *FUS*

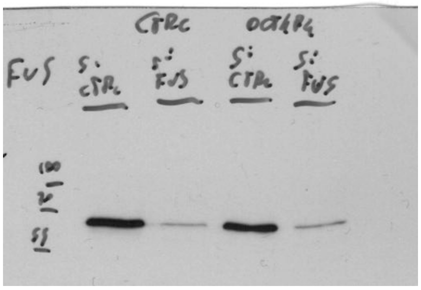

Fig. 4D *OCT4*

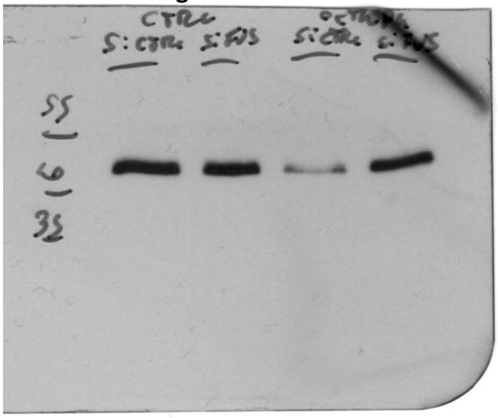

Fig. 4D *ACTIN*

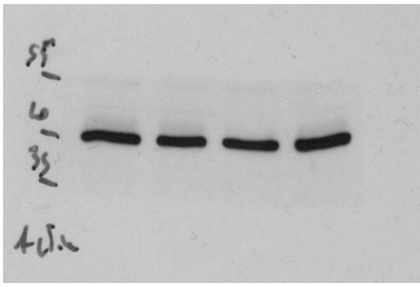

(c) Uncropped blots from Fig. 3. (d) Uncropped blots and gels from Figure 4. Images of the selected portions shown in Fig. 4B and Fig. 4C are indicated

Supplementary Fig. 6

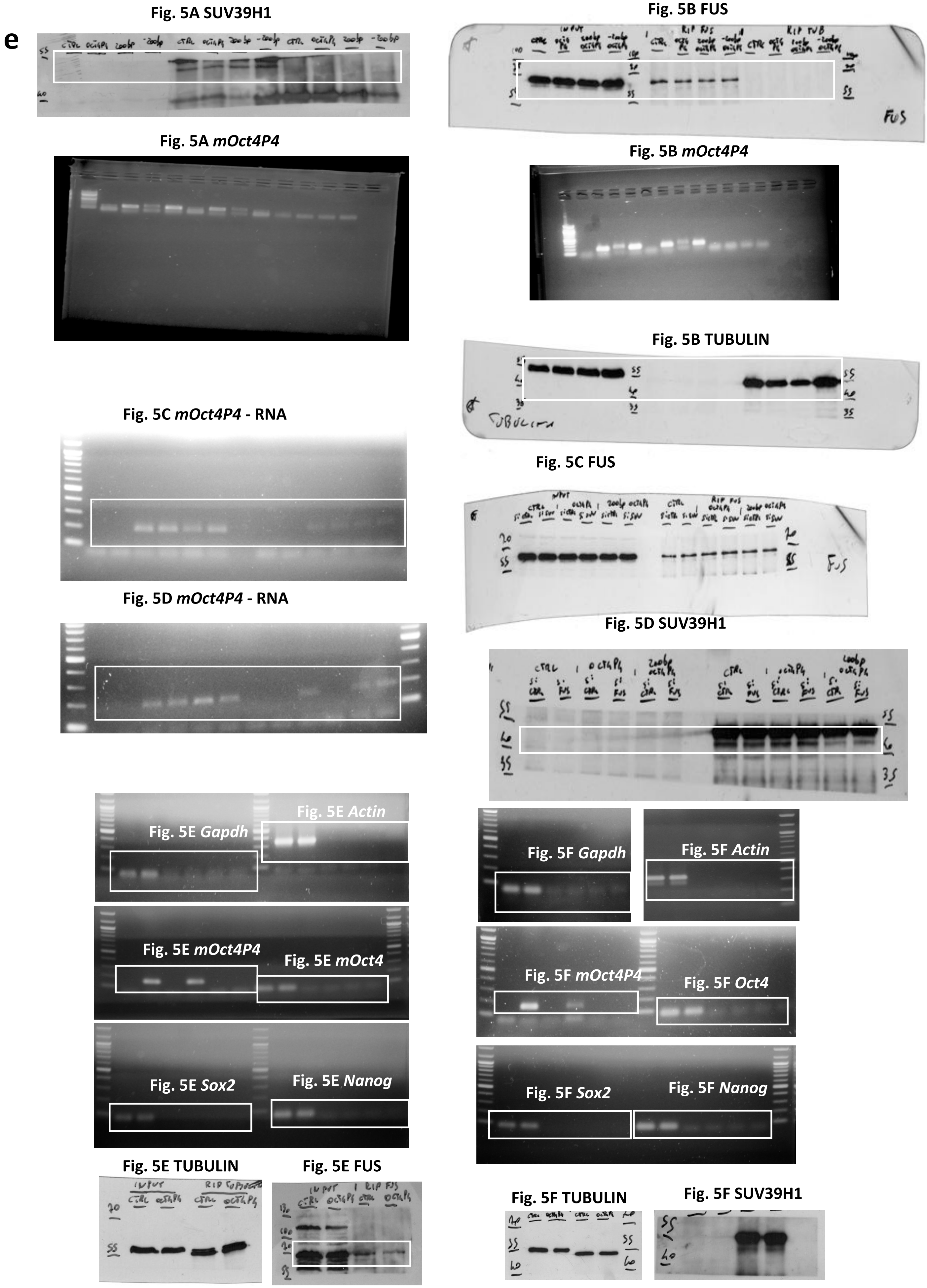

(e) Uncropped blots and gels from Figure 5. Images of the selected portions shown in Fig. 5A-F are indicated

Supplementary Fig. 6

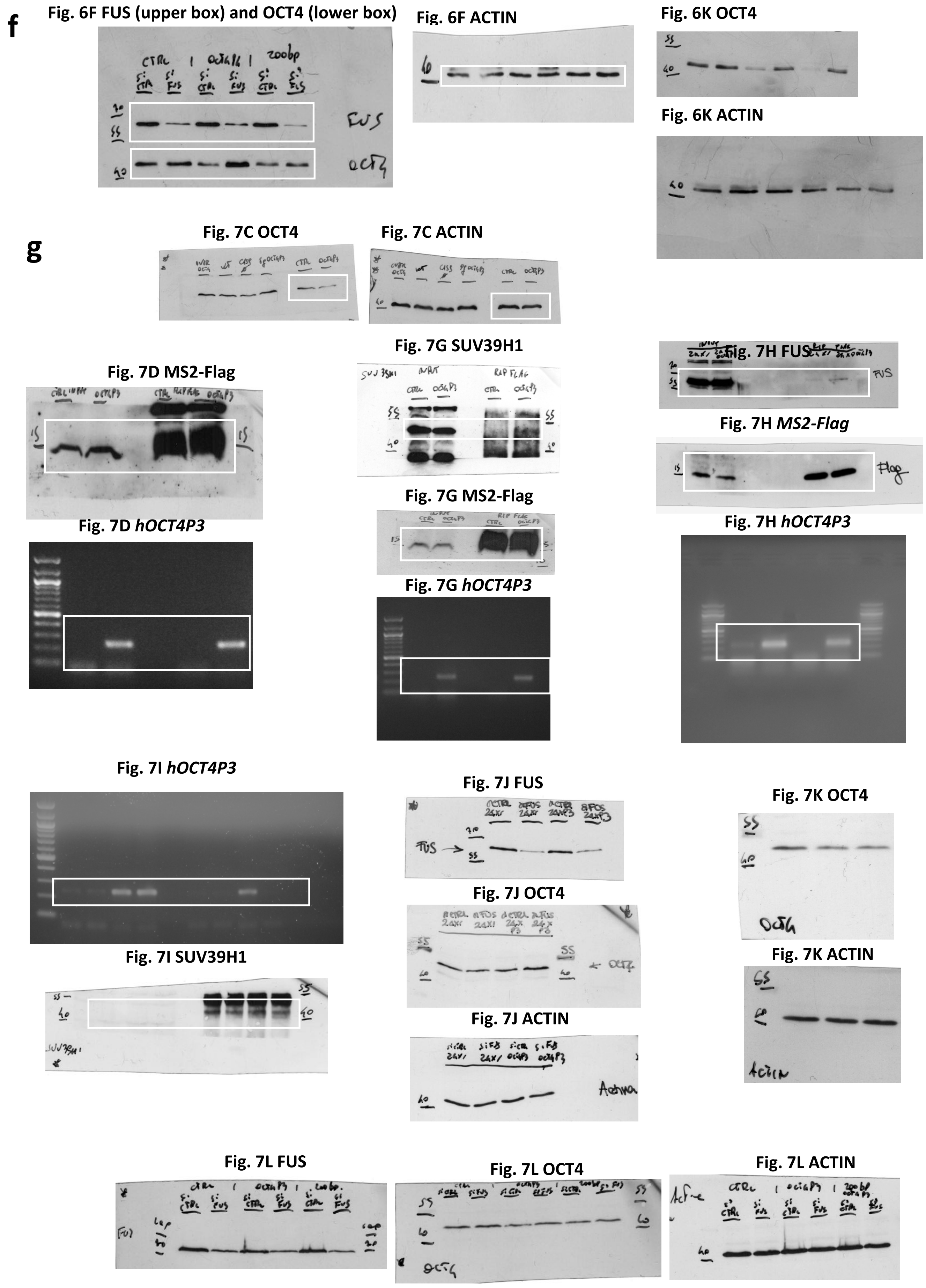

(f) Uncropped blots from Figure 6. Images of the selected portions shown in Fig. 6F are indicated. (g) Uncropped blots and gel from Figure 7. Images of the selected portions shown in Fig. 7I are indicated

Supplementary Fig. 6

h **Supplementary Fig. 1B OCT4 D3 and D10 short exposition**

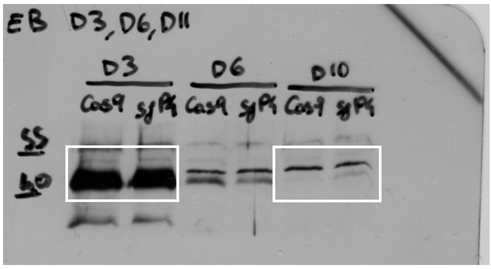

Supplementary Fig. 1B OCT4 D3, D10 long exposition

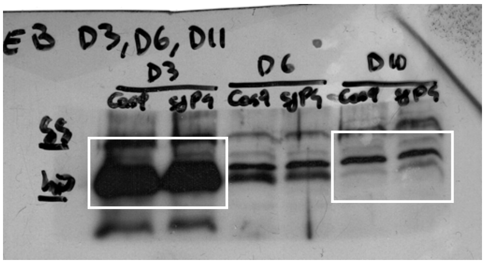

Supplementary Fig. 1B ACTIN T0, D3 and D10

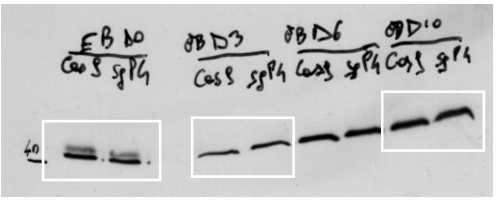

Supplementary Fig. 1B OCT4 T0 short exposition

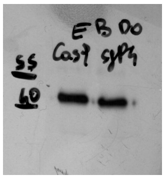

Supplementary Fig. 1B OCT4 T0 long exposition

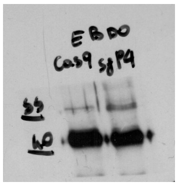

i **Supplementary Fig. 3D FUS**

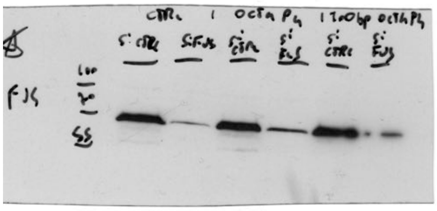

Supplementary Fig. 3D ACTIN

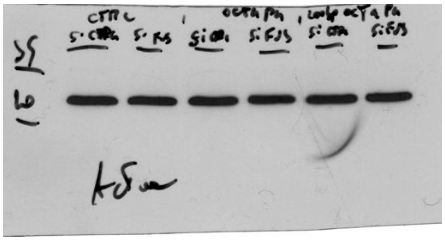

(h) Uncropped blots from Supplementary Figure 1. Images of the selected portions shown in Supplementary Fig. 1B are indicated. (i) Uncropped blots and gel from Supplementary Fig. 3D.

Supplementary Fig. 6

Band no.	Symbol	Description	Accession number	% Coverage	Score
1	MS2	Capsid protein Enterobacteria phage f2	P03611	8,53	47,11
2	PP1B	Peptidyl-prolyl cis-trans isomerase B	P24369	48,61	658,36
3	HMGB1	High mobility group box 1/2	P30681	19,53	75,29
	HMGB2		P63158	15,54	83,51
4	FUS	Fused in Sarcoma (FUS) RNA-binding protein	G3UXT7	43,85	93,69
5	eIF4B	Eukaryotic translation initiation factor 4B	Q8BGD9	21,44	635,12
6	NCL	Nucleolin	P09405	2,55	63,21

Supplementary Table 1. Mass spectrometry analysis of *mOct4P4-24xMS2* RIP eluates.

Proteins eluted from *mOct4P4* lncRNA RIP experiments and identified by mass spectrometry are indicated. Information on protein accession number, number of peptide matches, coverage and score values obtained from mass spectrometric analyses are indicated.

Supplementary Table 1

pLX sgRNA cloning	Forward/reverse cloning primers
PCR 1	F: AACTCGAGTGTACAAAAAAGCAGGCCTTTAAAG
	R: rc(GN$_{19}$)GGTGTTTCGTCCTTTCC
PCR 2	F: GN$_{19}$GTTTTAGAGCTAGAAATAGCAA
	R: AAAGCTAGCTAATGCCAACTTTGTACAAAGAAAGCTG

pLX sgRNA cloning	Forward/reverse cloning primers
mOct4P4 PCR 1	F: AACTCGAGTGTACAAAAAAGCAGGCCTTTAAAG
	R: CCAACTTGGGTGCCCAACTTCGGTGTTTCGTCCTTTCC
mOct4P4 PCR 2	F:GAAGTTGGGCACCCAAGTTGGGTTTTAGAGCTAGAAATAGCAA
	R: AAAGCTAGCTAATGCCAACTTTGTACAAAGAAAGCTG
hOCT4P3 PCR 1	F: AACTCGAGTGTACAAAAAAGCAGGCCTTTAAAG
	R: CCAGAAGTTCAAGACCAGCCTGGCGGTGTTTCGTCCTTTCC
hOCT4P3 PCR 2	F: GCCAGGCTGGTCTTGAACTTCTGGGTTTTAGAGCTAGAAATAGCAA
	R: AAAGCTAGCTAATGCCAACTTTGTACAAAGAAAGCTG

Supplementary Table 2. Oligonucleotides used for the establishment of pLX sgRNA vectors.

F, forward primer; R, reverse primer: For N$_{19}$ sequences see Supplementary Methods section.

Supplementary Table 2

**pLPC-mOct4P4-24xMS2 deletion constructs**	**Restriction sites and Forward/reverse cloning primers**
**Δ200**	F HindIII: GATTC **AAGCTT** GTCCCTAGGTGACCAACT
	R XhoI: GATTC **CTCGAG** AATGTGTACAGTGTGGTG
**Δ400**	F HindIII: GATTC **AAGCTT** GTCCCTAGGTGACCAACT
	R XhoI: GATTC **CTCGAG** CCCAAGCTGTTTGATGAT
**Δ600**	F HindIII: GATTC **AAGCTT** GTCCCTAGGTGACCAACT
	R XhoI: GATTC **CTCGAG** AGTTTACACAAGCTCTTG
**Δ800**	F HindIII: GATTC **AAGCTT** GTCCCTAGGTGACCAACT
	R XhoI: GATTC **CTCGAG** GGGACTTGGTTCCAGCTT
**Δ994**	F HindIII: GATTC **AAGCTT** GTCCCTAGGTGACCAACT
	R XhoI: GATTC **CTCGAG** TTATGGTAGGTGATGGCT
**3'UTR**	F XhoI: GATTC **CTCGAG** TAGAGAGGAAGATGAAGT
	R BglII: GATTC **AGATCT** TGTGTCCCAGGCTTTTTA

Supplementary Table 3. PCR oligos used for the generation of *mOct4P4* deletion constructs.
F, forward primer; R, reverse primer. Cloning sites are indicated in bold letters.

Supplementary Table 3

pLPC-200bp-Oct4P4-24xMS2 construct	Restriction sites and Forward/reverse cloning primers
**5'UTR**	F HindIII: GATTC **AAGCTT** GTCCCTAGGTGACCAACT
	R KpnI: GATTC **GGTACC** GGGGAAGTTGGGCACCCC
**200bp**	F KpnI: GATTC **GGTACC** GCGGCCCCTGCTGGAGAA
	R XhoI: GATTC **CTCGAG** CCCAAGCTGTTTGATGAT
**3'UTR**	F XhoI: GATTC **CTCGAG** TAGAGAGGAAGATGAAGT
	R BglII: GATTC **AGATCT** TGTGTCCCAGGCTTTTTA

Supplementary Table 4. PCR oligos used for the generation of the 200bp-*mOct4P4* expression vector.

F, forward primer; R, reverse primer. Cloning sites are indicated in bold letters.

**pLPC-(-200bp)-Oct4P4-24xMS2 construct**	**Restriction sites and Forward/reverse cloning primers**
**Upstream**	F HindIII: GATTC **AAGCTT** GTCCCTAGGTGACCAACT
	R XhoI: GATTC **CTCGAG** AGTTTACACAAGCTCTTG
**Downstream**	F BglII: GATTC **AGATCT** TGTGTCCCAGGCTTTTTA
	R BglII: GATTC **AGATCT** TGTGTCCCAGGCTTTTTA

Supplementary Table 5. PCR oligos used for the generation of the -200bp-*mOct4P4* expression vector.

F, forward primer; R, reverse primer. Cloning sites are indicated in bold letters.

Mouse	Sequence
200bp-mOct4P4 F	GAGGAAGCGGACAACAAT
200bp-mOct4P4 R	CTCTACCCAAGCTGTTTG
Fgf5 F	GTTTCCAGTGGAGCCCTT
Fgf5 R	GAGACACAGCAAATATTTCCAAAA
Fus F	CAGTCCTCGCGGCATCGCTT
Fus R	GGCCCCGTAGCTTTGAGTTGCT
Gapdh F	TTCACCACCATGGAGAAGGC
Gapdh R	CCCTTTTGGCTCCAC
Gdf3 F	TCAGCTTCTCCCAGACCAGGGTTT
Gdf3 R	CACACGCCCCGGTCCTGAAC
Nanog F	TTCTTGCTTACAAGGGTCTGC
Nanog R	AGAGGAAGGGCGAGGAGA
Oct4 F	CAGGGACACCTTTCCCAGGG
Oct4 R	TTTAAGAACAAAATGATGAG
Oct4P4 F	TGGCACCTGGCTTTAGACTTT
Oct4P4 R	CCAGGCCAACTTAGGGCATT
Sox2 F	TGCTGCCTCTTTAAGACTAGGG
Sox2 R	TCGGGCTCCAAACTTCTCT
sgOct4P4 F	GTTGGGCACCCCAAGTT
sgOct4P4 R	TCAAGTTGATAACGGACTAGCCT
Suv39h1	CGGATCACCGTGGAGAAT
Suv39h1 R	CACTCACAGCCAACAGCTACCT
**Human**	**Sequence**
ACTIN F	CCAACCGCGAGAAGATGA
ACTIN R	CCAGAGGCGTACAGGGATAG
FUS F	ATGGCCAGAGCCAGAACACAGG
FUS R	CGAGGTGCTGCTGGGAGCTG
KLF4 F	CAAGTCCCGCCGCTCCATTACCAA
KLF4 R	CCACAGCCGTCCCAGTCACAGTGG
NANOG F	ATGCCTCACACGGAGACTGT
NANOG R	AGGGCTGTCCTGAATAAGCA
OCT4A F	GGAGCCCTGCACCGTCA
OCT4A R	ATGGTCGTTTGGCTGAAT
OCT4P3 F	CTTCGGATTTCGCCTTCTCA
OCT4P3 R	GGGCACTAGCCCCACTCCAGT
sgOCT4P3 F	GCTGGTCTTGAACTTCTGGGT
sgOCT4P3 R	TCAAGTTGATAACGGACTAGCCT
SOX2 F	CCCACCTACAGCATGTCCTACTC
SOX2 R	TGGAGTGGGAGGAAGAGGTAAC

Supplementary Table 6. PCR oligos used for RT-qPCR analysis.

Species-specificity of primers is indicated. F, forward primer; R, reverse primer.

Murine	Primer sequence
Oct4 (primer pair A) F	TGCACCCCCTCCTCCTAATCC
Oct4 (primer pair A) R	CCCTAAACAAGTACTCAACCC
Dkk F	GGGAACCAGGGAAAGAGGA
Dkk R	GGGAAATA GGCACCCGATAA
**Human**	**Primer sequence**
OCT4 F	GAGGATGGCAAGCTGAGAAA
OCT4 R	CTCAATCCCCAGGACAGAAC
AChR F	CAACCAAAGCCCATGTCCTC
AChR R	AGGCACGCTACAGGGCTTC

Supplementary Table 7. PCR oligos used for qPCR analysis on ChIP eluates.

Species-specificity of primers is indicated. F, forward primer; R, reverse primer.

SUPPLEMENTARY METHODS

Generation of vectors:

To generate the pLX-sgOCT4P3 and pLx-sgOct4P4 we specifically amplified pLX-sgRNA plasmid (Addgene Plasmid #50662) by PCR. For oligonucleotide sequences see Supplementary table 2.

GN_{19} corresponds to the target sequence and rc(GN_{19}) to reverse complement of the target sequences: sgOct4P4 GN_{19}=GAAGTTGGGCACCCAAGTTGG; sgOCT4P3 GN_{19}=GCCAGGCTGGTCTTGAACTTCTGG. PCR1 and PCR2 amplicons were fused using a third PCR using Forward primer from PCR1 and Reverse primer from PCR2. PCR products were then cloned into pLX vector using NheI and XhoI restriction sites.

To generate the *hOCT4P3-24xMS2* construct, the *hOCT4P3* cDNA was PCR amplified using the following oligonucleotides: forward: GGGAATTC**AAGCTT** CAGCAAAGAACTAGAAGATAT; reverse: GGGAATTC**AGATCT** TTGTCTATCTACTGTGTTGA. PCR amplicon was cloned into into pLPC using HindIII and BglII restriction sites. Subsequently, 24 repeats of the MS2 stem loop RNA motif (obtained from pSL-MS2-24xMS2) were cloned downstream of the OCT4P3 cDNA insert.

pMSCV-HA-MS2-Flag was generated by PCR amplifying the Flag-MS2nls region in pCMV-Flag-MS2nls vector (forward primer: GGGAATTC**AGATCT**ATGGACTACAAAGACGATGA; reverse primer: GGGAATTC**CTCGAG**TTATACCTTTCTCTTCTTTT) and cloned into pMSCV vector using BamHI and XhoI.

pLPC-mOct4P4-24xMS2 deletion constructs (Δ200, Δ400, Δ600, Δ800, Δ994) were generated by PCR amplifying a specific mOct4P4 portion from pLPC-mOct4P4-24xMS2 construct and inserted together with the *mOct4P4* 3'UTR region into the pLPC-24xMS2 vector using HindIII and BglII restriction sites[1]. For primer sequences see Supplementary table 3.

For the generation of pLPC-200bp-mOct4P4-24xMS2 construct we PCR-amplified the 5'UTR, the 200-nucleotide region and the 3'UTR of pLPC-mOct4P4-24xMS2. Subsequently, PCR products were fused using the restriction sites indicated in Supplementary table 4. The obtained construct was cloned into the pLPC-24x vector via HindIII and BglII. pLPC-(-200bp)-mOct4P4-24xMS2 was generated by PCR amplifying the *mOct4P4* region upstream and downstream of the functionally relevant 200 bp region of pLPC-mOct4P4-24xMS2. The fused PCR products were cloned in pLPC-24xMS2 vector using HindIII and BglII (see Supplementary table 5).

RNA immunoprecipitation and mass spectrometry

MS2-flag/24xMS2 control and MS2-flag/mOct4P4-24xMS2 mESCs were used to perform anti-flag RNA immunoprecipitation (RIP). Briefly, cells were cross-linked in culture medium with 1 % formaldehyde for 10 minutes. Formaldehyde action was blocked in 125 mM glycine (1x PBS), cells were washed twice with cold PBS. Crosslinked cells were scraped in lysis buffer (50 mM Tris-HCl, pH 7.4; 1% Triton X-100; 1mM EDTA; 150 mM NaCl) supplemented with protease inhibitors and RNaseOUT (Invitrogen). Cell lysates were sonicated, centrifugated and the supernatants were pre-cleared with protein A/G PLUS-Agarose (sc-2003; Santa Cruz Biotechnology) for 1h at 4°C. The pre-cleared lysates were incubated with anti-FLAG M2 Magnetic Beads (M8823, Sigma-Aldrich) overnight at 4°C, following the manufacturer's suggestion. RNA-protein complexes were collected with magnetic beads and washed 10 times in lysis buffer. To elute the bound FLAG fusion complexes from the anti-FLAG M2 antibody, magnetic beads were incubated for 1h at 4°C with 50 µl 1xTBS solution containing 15µg FLAG Peptide (F3290, Sigma-Aldrich). The immunocomplexes proteins were resuspended in SDS-PAGE sample buffer and boiled for 15 minutes at 95°C.

The immunoprecipitates were separated by SDS-PAGE and a mass spectrometry-compatible silver staining (SilverQuest™ Kit, Invitrogen) or Coomassie Brilliant blue R-250 (42660, Sigma) was used to detect proteins in the PAA gel. Selected lanes were excised, de-stained, washed twice in 50% ACN with 50 mM ammonium bicarbonate and dehydrated in 100% ACN. In-gel reduction was performed by incubating gel slices in a 10 mM DTT, 100 mM ammonium bicarbonate solution for 30 min at 56°C followed by alkylation in 55 mM iodoacetamide, 100 mM ammonium bicarbonate for 20 min at room temperature. Protein samples were dehydrated again in 100% ACN and digested by rehydrating gel slices in 50 mM ammonium bicarbonate containing 4 ng/uL of trypsin (#V5111, Promega) overnight at 37°C. Tryptic peptides were desalted and concentrated using ZipTip mC18 pipet tips (Millipore) and co-eluted onto the MALDI target in 1 µL of α-cyano-4-hydroxycinnamic acid matrix (5 mg/mL in 50% ACN,0.1% TFA).

Mass spectra were acquired over a mass range of 800–4000 m/z (Nd:YAG laser at 355 nm, 40 Shots/Sub-Spectrum for 2,000 Total Shots/Spectrum) by reflectron positive mode on an Applied Biosystems 4,800 Proteomics Analyzer mass spectrometer (Applied Biosystems) and calibrated using a standard mixture (Mass Standards Kit, AB SCIEX). MS/MS spectra were acquired in positive mode (Nd:YAG laser at 355 nm, 40 Shots/Sub-Spectrum for 4,000 Total Shots/Spectrum) and MS/MS calibration was achieved by using the default calibration method.

Protein identifications were performed with the ProteinPilotTM software (version 2.0.1; Applied Biosystems) using the ParagonTM algorithm as the search engine. Each MS/MS spectrum was searched against an Uniprot/SwissProt database of mouse. The search parameters allowed for cysteine modification by iodoacetamide and biological modifications programmed in the algorithm (i.e., phosphorylations, semitryptic fragments, etc.). The detected protein threshold (ProtScore) in the software was set to 1.3 to achieve 95% confidence interval.

Western blotting and antibodies

An ice-cold lysis buffer containing 10 mM Tris-HCl, pH 7.4, 100 mM NaCl, 1 mM PMSF, 20 mM β–glycerophosphate (Sigma-Aldrich), 1 mM Na3VO4, 1 mM Na2F, 5 mM EDTA, protease inhibitor cocktail (Sigma) and 1% Triton X-100 (Sigma-Aldrich) was used to prepare OVCAR-3 and mESCs lysates. Western blotting was carried out according to standard procedures. The following primary antibodies were used for western blotting: rabbit polyclonal anti-Oct4 (ab19857, abcam), rabbit polyclonal anti-actin (A2066, Sigma), mouse monoclonal anti-Flag (clone M2, F1804), mouse monoclonal anti-KMT1A/Suv39h1 (ab12405) and rabbit polyclonal anti-TLS/Fus (ab23439, abcam). Secondary antibodies coupled to horseradish peroxidase were obtained from Sigma (anti-rabbit IgG peroxidase conjugate A-6154; anti-mouse IgG peroxidase conjugate A-4416). ImageJ software (http://imagej.nih.gov/ij/) was used to quantify protein bands.

RNA extraction and real-time PCR

Total and immunoprecipitated RNA were extracted with the Qiazol lysis reagent (Qiagen), subjected to DNase treatment (Qiagen) and subjected to reverse transcription (Quantitect reverse transcription kit; Qiagen). The obtained cDNA was used for quantitative real-time PCR (SYBR Green Universal PCR Master Mix, Applied Biosystems) on a StepOnePlus real-time PCR machine (Applied Biosystems). Expression levels were normalized against Gapdh.

Nuclear and cytoplasmic RNA fractions were obtained by collecting cells in lysis buffer (10mM NaCl, 20mM MgCl, 10mM Tris-Cl, pH 7.8, 5mM DTT, 0.5% NP-40). After incubation on ice for 5 minutes, nuclei were pelleted by centrifugation at 8000 rpm for 5 minutes at 4°C. Obtained nuclei were washed and resuspended in lysis buffer. The cytoplasmic fraction was collected and cleared by centrifugation. Nuclear and cytoplasmic fractions were subjected to protease treatment for 20 minutes at 37°C in proteinase K solution (300 mM NaCl, 0.2 M Tris-Cl, pH7.5, 25mM EDTA, 2% SDS

and 0.1 mg/ml proteinase K). Subsequently, RNA was purified using the Qiazol lysis reagent (Qiagen). Quantitative RT-PCR primer sequences are reported below (Supplementary table 6)

Chromatin immunoprecipitation

Cells were cross-linked in culture medium with 1% formaldehyde for 15 min, neutralized using 125mM glycine in PBS and washed in PBS. Nuclei were obtained by lysing scraped cells in hypotonic buffer (5mM Pipes pH 6.8, 85mM KCl, 0.5% NP-40 and protease inhibitors), followed by centrifugation. Nuclei were resuspended in RIPA 100mM buffer (20mM Tris-HCl pH 7.5, 100mM NaCl, 1mM EDTA, 0.5% NP-40, 0.5% Na-Deoxycholate, 0.1% SDS supplemented with protease inhibitors). Chromatin was sonicated to 500-800 bp fragment size and pre-cleared for 1h at 4°C with protein A/G PLUS-Agarose beads (Santa Cruz, sc-2003). Agarose was removed by centrifugation and an aliquot of supernatant was taken as input. Chromatin was immunoprecipitated overnight at 4 °C with the following antibodies: mouse monoclonal anti-Flag M2, clone M2 (2.5 mg/ml, F1804) and anti-H3K9me3 (Upstate), mouse monoclonal anti-KMT1A/Suv39h1 (ab12405) and rabbit polyclonal anti-TLS/Fus (ab23439, abcam). DNA–protein complexes were recovered with protein A/G PLUS Agarose beads and washed with RIPA 100mM buffer, RIPA 250mM buffer (20mM Tris-HCl pH 7.5, 250mM NaCl, 1mM EDTA, 0.5% NP-40, 0.5% Na-Deoxycholate and 0.1% SDS), LiCl solution (10mM Tris-HCl pH 8.0, 1mM EDTA, 250mM LiCl, 0.5% NP-40 and 0.5% Na-Deoxycholate) and 1xTris-EDTA (TE). RNase treatment was performed in 1xTE for 30 min at 37°C. Crosslinking was reversed by overnight incubation at 68°C after adding an equal volume of proteinase K solution (200mM NaCl, 1% SDS and 0.3 mg ml^{-1} proteinase K). Samples were resuspended in ddH2O after phenol/chloroform extraction and ethanol precipitation. Co-immunoprecipitated DNA was analyzed by quantitative RT-PCR. ChIP data of Oct4 promoter regions were normalized against input and the unrelated Dkk or AChR control gene. Primers are listed in Supplementary table 7.

Co-Immunoprecipitation

For co-immunoprecipitation experiments cells were lysed in RIPA buffer (50 mM Tris-Cl, pH 7.5, 1% Nonidet P-40 (NP-40), 0.5% sodium deoxycholate, 0.05% SDS, 1 mM EDTA,150 mM NaCl) with protease inhibitors. Samples were pre-cleared with protein A/G PLUS-Agarose beads (sc-2003; Santa Cruz Biotechnology) for 1 h followed by centrifugation. Precleared supernatants were incubated overnight at 4 °C with the following antibodies: rabbit polyclonal anti-TLS/Fus (ab23439, abcam) or mouse monoclonal anti-KMT1A/Suv39h1 (2.5 mg/ml, ab12405, Abcam). After 3 h of

incubation with protein A/G PLUS-Agarose, immunoprecipitates were washed three times in RIPA buffer, resuspended in sample buffer, and analyzed by western blotting.

Quantitative determination of *hOCT4P3, OCT4 and DAXX* RNA molecules
To estimate the copy number of endogenous *hOCT4P3, OCT4 and DAXX* transcripts, vectors containing subcloned fragments of *hOCT4P3, OCT4 and DAXX* were used as internal standards in quantitative PCR experiments [2]. 0,2 μg of total RNA from Ovcar-3 cells was subjected to reverse transcription using the SuperScript™ IV VILO™ Master Mix with ezDNase™ Enzyme kit (Thermo Fisher Scientific) according to the manufacturer's protocol. Equal volumes (5μl) of target cDNA and serially dilutions of vectors (mol/ml; external standard) were used in quantitative RT-PCR in order to determine the molar range of the 3 target RNAs. To more precisely define the number of *hOCT4, hOCT4P3* molecules in 5μl of cDNA, limited molar dilutions of vectors (internal standard) were added to target cDNA in each quantitative RT-PCR reaction. This enables us to determine the number of *OCT4/hOCT4P3/DAXX* mRNA molecules in each reaction tube. The number of molecules obtained for h*OCT4P3, OCT4 and DAXX* was expressed as molecules per μg of total RNA used for reverse transcription.

SUPPLEMENTARY REFERENCES:

1. Scarola, M. *et al.* Epigenetic silencing of Oct4 by a complex containing SUV39H1 and Oct4 pseudogene lncRNA. *Nat. Commun.* **6**, (2015).
2. Adamski, M. G., Gumann, P. & Baird, A. E. A method for quantitative analysis of standard and high-throughput qPCR expression data based on input sample quantity. *PLoS One* **9**, e103917 (2014).
