# Supplementary material for: FUS-dependent loading of SUV39H1 to OCT4 pseudogene-lncRNA programs a silencing complex with OCT4 promoter specificity
Source: Commun Biol. 2020 Oct 30;3:632. doi: 10.1038/s42003-020-01355-9 (PMC7603346; doi:10.1038/s42003-020-01355-9)
Supplement: Supplementary file 3 — Description of Additional Supplementary Files [file 42003_2020_1355_MOESM3_ESM.pdf]

Description of Additional Supplementary Files

File Name: Supplementary Movie 1
Description: Contractile cardiomyocyte structures in differentiated dCas9 embryonic stem cells.

File Name: Supplementary Movie 2
Description: Reduced formation of contractile cardiomyocyte structures in differentiated dCas9/sgOct4P4 embryonic stem cells.

File Name: Supplementary Data 1
Description: Mass spectrometry results obtained from mOct4P4-24xMS2 lncRNA RIP experiments using mESCs. Identified peptides and related data obtained from protein bands indicated in main figure 4a are shown.
