# Supplementary material for: FUS-dependent loading of SUV39H1 to OCT4 pseudogene-lncRNA programs a silencing complex with OCT4 promoter specificity
Source: Commun Biol. 2020 Oct 30;3:632. doi: 10.1038/s42003-020-01355-9 (PMC7603346; doi:10.1038/s42003-020-01355-9)
Supplement: Supplementary file 7 — Reporting Summary [file 42003_2020_1355_MOESM7_ESM.pdf]

nature research

Corresponding author(s): Benetti Roberta
Schoeftner Stefan

Last updated by author(s): 2020/09/23

Reporting Summary

Nature Research wishes to improve the reproducibility of the work that we publish. This form provides structure for consistency and transparency in reporting. For further information on Nature Research policies, see our Editorial Policies and the Editorial Policy Checklist.

Statistics

For all statistical analyses, confirm that the following items are present in the figure legend, table legend, main text, or Methods section.

n/a	Confirmed	
☐	☒	The exact sample size (*n*) for each experimental group/condition, given as a discrete number and unit of measurement
☐	☒	A statement on whether measurements were taken from distinct samples or whether the same sample was measured repeatedly
☐	☒	The statistical test(s) used AND whether they are one- or two-sided<br>*Only common tests should be described solely by name; describe more complex techniques in the Methods section.*
☒	☐	A description of all covariates tested
☒	☐	A description of any assumptions or corrections, such as tests of normality and adjustment for multiple comparisons
☐	☒	A full description of the statistical parameters including central tendency (e.g. means) or other basic estimates (e.g. regression coefficient) AND variation (e.g. standard deviation) or associated estimates of uncertainty (e.g. confidence intervals)
☐	☒	For null hypothesis testing, the test statistic (e.g. *F*, *t*, *r*) with confidence intervals, effect sizes, degrees of freedom and *P* value noted<br>*Give P values as exact values whenever suitable.*
☒	☐	For Bayesian analysis, information on the choice of priors and Markov chain Monte Carlo settings
☒	☐	For hierarchical and complex designs, identification of the appropriate level for tests and full reporting of outcomes
☒	☐	Estimates of effect sizes (e.g. Cohen's *d*, Pearson's *r*), indicating how they were calculated

Our web collection on statistics for biologists contains articles on many of the points above.

Software and code

Policy information about availability of computer code

Data collection	Results from western blots of protein extracts, immunoprecipitates and RNA immunoprecipitates were obtained with ECL western blotting reagents. Quantitative RT-PCR data was obtained with a StepOnePlus real time PCR machine (Applied Biosystems). Mass spectrometry data were obtained with an Applied Biosystems 4,800 Proteomics Analyzer mass spectrometer (Applied Biosystems)
Data analysis	The following software was used in this study:<br>ProteinPilotTM software (version 2.0.1; Applied Biosystems) using the ParagonTM algorithm as the search engine for mass spectrometry analysis. ImageJ for quantification of western blots. Microsoft Excel for analysis of quantitative RT-PCR data. Graph Pad Prism for statistical analysis and graph plotting;

For manuscripts utilizing custom algorithms or software that are central to the research but not yet described in published literature, software must be made available to editors and reviewers. We strongly encourage code deposition in a community repository (e.g. GitHub). See the Nature Research guidelines for submitting code & software for further information.

Data

Policy information about availability of data

All manuscripts must include a data availability statement. This statement should provide the following information, where applicable:

- Accession codes, unique identifiers, or web links for publicly available datasets
- A list of figures that have associated raw data
- A description of any restrictions on data availability

All data generated or analyzed during this study are included in this published article and related supplementary information files.
Source data of blots and gels are shown in Supplementary Figure 6.

Field-specific reporting

Please select the one below that is the best fit for your research. If you are not sure, read the appropriate sections before making your selection.

☒ Life sciences ☐ Behavioural & social sciences ☐ Ecological, evolutionary & environmental sciences

For a reference copy of the document with all sections, see nature.com/documents/nr-reporting-summary-flat.pdf

Life sciences study design

All studies must disclose on these points even when the disclosure is negative.

Sample size	Sample size was chosen according to accepted standards in the field. Sample size was not pre-determined using statistics tools. As indicated in the figures of the manuscript, the size of statistically analyzed independent biological samples was "3" unless differently specified. Statistical analysis (as described in respective figure legends) was used to calculate significance of obtained results. Precise p-values are indicated in all figures.
Data exclusions	No data were escluded in the analysis
Replication	All experiments were reproducible
Randomization	No randomization; All experiments are based on gain or loss of function experiments with appropriate controls
Blinding	No blinding was applied. For critical data, 2 experimenters carried out analysis of biological replicates

Reporting for specific materials, systems and methods

We require information from authors about some types of materials, experimental systems and methods used in many studies. Here, indicate whether each material, system or method listed is relevant to your study. If you are not sure if a list item applies to your research, read the appropriate section before selecting a response.

Materials & experimental systems

n/a	Involved in the study	
☐	☒	Antibodies
☐	☒	Eukaryotic cell lines
☒	☐	Palaeontology and archaeology
☒	☐	Animals and other organisms
☒	☐	Human research participants
☒	☐	Clinical data
☒	☐	Dual use research of concern

Methods

n/a	Involved in the study	
☒	☐	ChIP-seq
☒	☐	Flow cytometry
☒	☐	MRI-based neuroimaging

Antibodies

Antibodies used	1) rabbit polyclonal anti-Oct4 (ab19857, Abcam) 2)rabbit polyclonal anti-actin (A2066, Sigma-Aldrich) 3) mouse monoclonal anti-Flag (clone M2, F1804, Sigma-Aldrich) 4) mouse monoclonal anti-KMT1A/Suv39h1 (ab12405, Abcam) and 5) rabbit polyclonal anti-TLS/Fus (ab23439, abcam). Secondary antibodies coupled to horseradish peroxidase were obtained from Sigma (anti-rabbit IgG peroxidase conjugate A-6154; anti-mouse IgG peroxidase conjugate A-4416 6) rabbit polyclonal anti-H3K9me3 (Upstate 07-442)<br>7) mouse monoclonal anti-HA antibody, clone HA-7 (Sigma H9658)
Validation	1) anti-Oct4 (ab19857, abcam): https://www.abcam.com/oct4-antibody-ab19857.html<br>Validation: reduction of OCT4 protein expression in gain of function mOct4P4 lncRNA mESCs (Fig. 2e); increased OCT4 protein levels in dCas9 sgOCT4P3 OVCAR3 cells (Fig 1k); down-regulation of OCT4 during mESC differentiation (Suppl. Fig. 6h); see also Comisso et al. 2017, doi: 10.1038/onc.2017.20.<br><br>2) anti-Actin (A2066, Sigma-Aldrich): https://www.sigmaaldrich.com/catalog/product/sigma/a2066?lang=it&region=IT&gclid=CjwKCAjw5Kv7BRBSEiwAXGDEleVkW1pq_LGY66vBONbQ-9ewMs-iCFo42sBptaCRXoAQzWrFq5tXQhoCdUYQAvD_BwE<br>Validation: was used as loading control in western blots<br><br>3) mouse monoclonal anti-Flag (clone M2, F1804, Sigma-Aldrich): https://www.sigmaaldrich.com/catalog/product/sigma/f1804?lang=it&region=IT&gclid=CjwKCAjw5Kv7BRBSEiwAXGDEleAy3hK0u1MiAR1oYyL_jGtDXsStAsTxevVpe52ROpHFbFFPsTOTyhoCw4EQAvD_BwE

Validation: Immunoprecipitation in Fig. 4c; see also Scarola at al. 2015, doi10.1038/ncomms8631

4) mouse monoclonal anti-KMT1A/Suv39h1 (ab12405, abcam): https://www.abcam.com/kmt1a--suv39h1-antibody-441-ab12405.html
Validation: specific immunoprecipitation of SUV39H1 using the Abcam ab12405 antibody in Supplementary figure 3a, b

5) anti-TLS/Fus (ab23439, abcam): https://www.abcam.com/tlsfus-antibody-ab23439.html
Validation: reduced FUS protein levels after siRNA mediated knock-down of FUS (Fig. 6f and Supplementary figure 3d)

6) rabbit polyclonal anti-H3K9me3 (Upstate 07-442)
Validation: specific chromatin immunoprecipitation in Fig. 2g and Fig. 3g. see also Scarola et al. doi10.1038/ncomms8631

7) mouse monoclonal anti-HA antibody, clone HA-7 (Sigma H9658): https://www.sigmaaldrich.com/catalog/product/sigma/h9658?lang=it&region=IT
Validation: no signal in western blots using extracts of mESC without ectopic expression of HA-tagged dCAS9 (Fig. 1d); no immunoprecipitation in RIP control experiments (Fig. 5a)

Eukaryotic cell lines

Policy information about cell lines

Cell line source(s)	-OVCAR-3 cells were obtained from ATCC (NIH:OVCAR-3 [OVCAR3] (ATCC® HTB-161TM).<br>-TOV-112D cells were obtained from ATCC (ATCC CRL-11731)<br>-SK-OV-3 cells were obtained from ATCC (ATCC HTB-77)<br>-COAV3 cells: obtained from ATCC (ATCC HTB-75)<br>mESCs: obtained from G.J. Hannon (Murchison E. P., Partridge J. F., Tam O. H., Cheloufi S. & Hannon G. J. Characterization of Dicer-deficient murine embryonic stem cells. Proc. Natl Acad. Sci. USA 102, 12135–12140 (2005))<br>-Primary mouse embryonic fibroblasts (pMEFs) were generated in-house from 13.5 d.p.c. C57BL/6 mouse embryos
Authentication	Cells used have not been cultured for longer than 6 months
Mycoplasma contamination	All used cell lines tested negative for mycoplasma contamination in regular intervals
Commonly misidentified lines<br>(See ICLAC register)	No misidentified cell lines were used in this study
